# The effectiveness of the Schroth method of physical therapy for treating an adult with adolescent idiopathic scoliosis (AIS) in an outpatient clinic in the United States with third-party payer constraints: a case report

**DOI:** 10.1186/1748-7161-8-S2-O10

**Published:** 2013-09-18

**Authors:** Berdishevsky Hagit

**Affiliations:** 1 Hospital for Special Surgery, New York, NY, USA

## Background

Very few studies examine the efficacy of outpatient Schroth therapy for the management of scoliosis, especially in countries such as the U.S. where a third-party payer constrains the therapy duration to an average of 1-3 sessions. Study patient description: double major scoliosis, thoracic Cobb angle 38° and lumbar Cobb angle 24°. Angle of Trunk Rotation (ATR) was 13° in the thoracic and 10° in the lumbar, quality of life score was 2.28 (SRS 22 questionnaire), body image was 1 (Trunk Appearance Perception Scale (TAPS)), pain score was 7 using the Visual Analog Scale (VAS), force vital capacity (FVC) was 2.8 liters, chest expansion was 3.8 cm in the subaxillary, 2.5 cm in the nipple line and 0.5cm around the waist. Trunk deviation from the central sacral line was 2 cm to the left.

## Purpose

The aim of this study was to examine the effects of Schroth therapy on a 26 year old female with AIS.

## Methods

The physical therapy regimen included 1-hour Schroth exercise sessions twice per week for four weeks followed by one session each week for 20 additional weeks. In addition, a home exercise program, which consisted of 30-minute sessions five days per week, was recommended. All tests and measurements were recorded before and after treatment.

## Results

After a 6-month treatment period, the patient experienced significant and measurable improvement. The Cobb angle was reduced to 27.6° in the thoracic and 19° in the lumbar, the ATR had decreased to 8° in the thoracic and 6° in the lumbar, quality-of-life score and self-image showed improvement with a score of 4.02 on the SRS 22 and 3 on the TAPS. Pain score diminished to 2. Trunk deviation improved by 1 cm, FVC increased to 3.2 liters and chest expansion to 4.5 cm, 3.5 cm and 1.5 cm at the measured locations. In addition, the patient's strength improved and she felt more comfortable with her appearance and reported satisfaction with the results.

## Conclusions and discussion

These preliminary findings suggest that out-patient physical therapy utilizing the Schroth method may be a successful alternative to the traditional methods of treating scoliosis.
